# Dehydroandrographolide, an iNOS inhibitor, extracted from from *Andrographis paniculata* (Burm.f.) Nees, induces autophagy in human oral cancer cells

**DOI:** 10.18632/oncotarget.5036

**Published:** 2015-09-03

**Authors:** Ming-Ju Hsieh, Chiao-Wen Lin, Hui-Ling Chiou, Shun-Fa Yang, Mu-Kuan Chen

**Affiliations:** ^1^ Cancer Research Center, Changhua Christian Hospital, Changhua 500, Taiwan; ^2^ School of Optometry, Chung Shan Medical University, Taichung 40201, Taiwan; ^3^ Institute of Medicine, Chung Shan Medical University, Taichung 40201, Taiwan; ^4^ Institute of Oral Sciences, Chung Shan Medical University, Taichung 40201, Taiwan; ^5^ Department of Dentistry, Chung Shan Medical University Hospital, Taichung 40201, Taiwan; ^6^ School of Medical Laboratory and Biotechnology, Chung Shan Medical University, Taichung 40201, Taiwan; ^7^ Department of Clinical Laboratory, Chung Shan Medical University Hospital, Taichung 40201, Taiwan; ^8^ Department of Medical Research, Chung Shan Medical University Hospital, Taichung 40201, Taiwan; ^9^ Department of Otorhinolaryngology-Head and Neck Surgery, Changhua Christian Hospital, Changhua 500, Taiwan

**Keywords:** dehydroandrographolide, autophagy, p53, MAPK, oral cancer

## Abstract

Autophagy, which is constitutively executed at the basal level in all cells, promotes cellular homeostasis by regulating the turnover of organelles and proteins. Andrographolide and dehydroandrographolide (DA) are the two principle components of *Andrographis paniculata* (Burm.f.) Nees. and are the main contributors to its therapeutic properties. However, the pharmacological activities of dehydroandrographolide (DA) remain unclear. In this study, DA induces oral cancer cell death by activating autophagy. Treatment with autophagy inhibitors inhibited DA-induced human oral cancer cell death. In addition, DA increased LC3-II expression and reduced p53 expression in a time- and concentration-dependent manner. Furthermore, DA induced autophagy and decreased cell viability through modulation of p53 expression. DA-induced autophagy was triggered by an activation of JNK1/2 and an inhibition of Akt and p38. In conclusion, this study demonstrated that DA induced autophagy in human oral cancer cells by modulating p53 expression, activating JNK1/2, and inhibiting Akt and p38. Finally, an administration of DA effectively suppressed the tumor formation in the oral carcinoma xenograft model *in vivo*. This is the first study to reveal the novel function of DA in activating autophagy, suggesting that DA could serve as a new and potential chemopreventive agent for treating human oral cancer.

## INTRODUCTION

Oral squamous cell carcinoma (OSCC) is the most common head and neck cancer and has a poor prognosis and low survival rate. [1] Chemotherapy is the suggested treatment for advanced-stage cancers. Many vegetables, fruits, and grains offer substantial protection against various cancers. [2–4] Increasing focus is on providing a scientific basis for using these agents as a preventive strategy for people with a high risk of cancer. Therefore, inducing the death of cancer cells by using chemotherapeutic agents may facilitate achieving successful chemotherapy treatment in patients.

Autophagy is a major intracellular degradation mechanism that operates under stress conditions and can promote survival during starvation or lead to programmed type II cell death under specific conditions (e.g., apoptosis inhibition). [5–7] Autophagy is a multifaceted process, and alterations in autophagic signaling pathways are frequently observed in cancer. However, the role of autophagy in cancer remains controversial. [8, 9] One hypothetical mechanism is that autophagy promotes tumor cell survival in response to various stresses. [10] Inhibiting autophagy has been determined to promote tumorigenesis, whereas enhancing autophagy in established tumors has been reported to promote cell survival. [11, 12] Furthermore, autophagy spatially and temporally regulates tumor development by suppressing tumor growth through the regulation of cell proliferation in the early stages of tumorigenesis. [13] Therefore, reduction in the rate of autophagy contributes to tumor formation and growth as a result of the breakdown of tumor cells following autophagy-related cell death, leading to tumor cell survival. [14] Recently, the role of autophagy in cancer development and therapy has received extensive attention. [15–17] Certain reagents can kill cancer cells through some non-apoptotic pathways and evade chemoresistance, which makes such reagents promising candidate molecules for treating drug-resistant cancers. When treated with these reagents, tumor cells with drawback in apoptosis undergo autophagy, and inhibiting autophagy causes tumor cells death through alternative mechanisms. [18, 19]

The mechanism of autophagy is a highly conserved process; genetic analyses in yeast have identified several AuTophaGy-related (ATG) genes, many of which have mammalian orthologs. ATG products are implicated in autophagosome formation and associated pathways. [20] Upon autophagy induction, light chain 3 (LC3)-I, the mammalian ortholog of yeast Atg8, conjugates with phosphatidylethanolamine to form the autophagosome-associated LC3-II. LC3-II levels (relative to actin or tubulin loading controls) correlate with autophagosome numbers. [21] The classical pathway regulating mammalian autophagy involves serine or threonine kinases, the mammalian target of rapamycin (mTOR). [22] Various signals, such as growth factors, amino acids, and energy status, regulate autophagy through the mTORC1 pathway. [23]

*In vitro* studies of mammalian cells have suggested that ROS regulate autophagy in various cell lines, because exogenous oxidative stressors induce autophagy. For example, H_2_O_2_ and 2-methoxyestradiol induce autophagy in transformed HEK293 cells, U87 cells, HeLa cells, and astrocytes. [24, 25] TNF-alpha induces autophagy in EW7 cells in a ROS-dependent manner, and H_2_O_2_ scavenging inhibits starvation-induced autophagy. [26] Similarly, the endotoxin LPS induces autophagy in an H_2_O_2_-dependent manner in cardiomyocytes. [27] In addition, nitric oxide (NO), a potent cellular messenger, inhibits autophagosome synthesis through several mechanisms. NO impairs autophagy by inhibiting the activity of S-nitrosylation substrates, JNK1, and IKKβ. Overexpression of nNOS, iNOS, or eNOS impairs autophagosome formation primarily through the JNK1–Bcl-2 pathway. Conversely, NOS inhibition enhances the clearance of autophagic substrates. [28] These results suggest that autophagy induction may trigger programmed type II cell death by inhibiting NOS expression.

*Andrographis paniculata* (Burm.f.) Nees (family, Acanthaceae), which is grown widely in many Asian countries, has been shown to possess various pharmacological properties such as anticancer, anti-HIV, anti-influenza virus, and cardioprotective properties. [29–31] The reported primary active ingredients of *A. Paniculata* are several diterpene lactones, flavonoids, and polyphenols. [32, 33] Two principle components, namely, andrographolide and dehydroandrographolide (DA), are believed to be the main contributors to its therapeutic properties. Previous studies have reported that DA inhibits LPS-induced oxidative stress by inactivating iNOS. [34] In addition, DA inhibits viral DNA replication. [35] These studies confirm that DA is an iNOS inhibitor and an antiinflammatory [36] and antiviral agent. However, the pharmacological properties of DA remain unclear. The aim of this study was to characterize the effects of DA on human oral cancer cells and elucidate the underlying molecular mechanism responsible for autophagy in DA-treated oral cancer cells.

## RESULTS

### Cytotoxic effects of DA on human oral cancer cell lines

**Figure 1:**
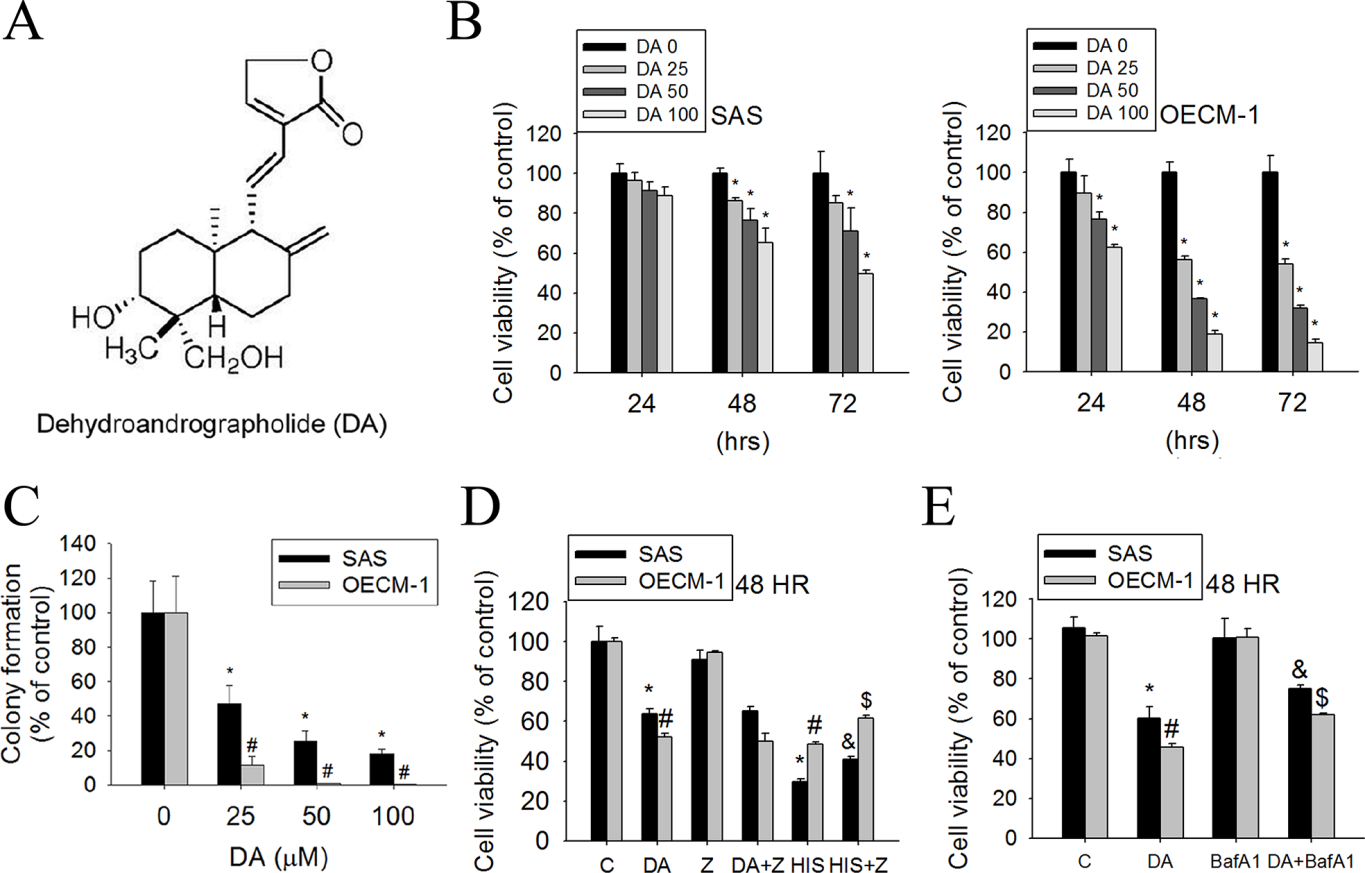
Effect of DA on cell viability in SAS and OECM-1 cell lines **A.** Structure of DA. **B.** Cell viability of SAS and OECM-1 cells (2 × 10^4^ cells/well of 96-well plate) cultured in presence of various concentrations of DA (0–100 μM) for 24, 48 and 72 h, as analyzed by MTT assay. **C.** Equal numbers of cells from the DA-treated SAS and OECM-1 cell pools were plated and stained as described in the text. The number of colonies was counted under a dissecting microscope. The data show the relative colony number, and the number of cell lines without DA treatment was set at 100%. Results are shown as mean ± SE. *p < 0.05, compared with the SAS (0 μM). #*P* < 0.05, compared with the OECM-1 (0 μM). **D.** SAS and OECM-1 cells (5 × 10^4^ cells/well of 24-well plate) were treated with DA (100 μM) or Hispolon (10 μM) in the presence or absence of Z-VAD-FMK (20 μM) for 48 h and analyzed by MTT assay. Results are shown as mean ± SE. *P < 0.05, compared with the SAS (0 μM). #*P* < 0.05, compared with the OECM-1 (0 μM). &*P* < 0.05, compared with the SAS (Hispolon, 10 μM). $*P* < 0.05, compared with the OECM-1 (Hispolon, 10 μM). **E.** SAS and OECM-1 cells (5 × 10^4^ cells/well of 24-well plate) were treated with DA (100 μM) in the presence or absence of autophagy inhibitor BafA1 (1 nM) for 48 h, as analyzed by MTT assay. Results are shown as mean ± SE. *P < 0.05, compared with the SAS (0 μM). #*P* < 0.05, compared with the OECM-1 (0 μM). &*P* < 0.05, compared with the SAS treated with DA (100 μM). $*P* < 0.05, compared with the OECM-1 treated with DA (100 μM).

The chemical structure of DA is shown in Figure 1A. To assess the effects of DA on cell viability, SAS and OECM-1 cells were treated with DA at various concentrations (0–100 μM) for 24, 48, and 72 h, and then analyzed using the MTT assay. DA substantially reduced the cell viability after 48 h of treatment in SAS and OECM-1 cells compared with untreated cells (Figure 1B). In particular, DA inhibited cell viability; this inhibition was observed within 24 h in OECM-1 cells. To further investigate the anti–cell-growth activity of DA, a clonogenic assay was performed to determine the long-term effect of DA treatment on oral cancer cells. DA (25 μM) significantly inhibited the colony-formation ability of SAS and OECM-1 cells (Figure 1C). To clarify the relevance of DA-induced cell death, Z-VAD-FMK (a broad-spectrum caspase inhibitor) and an autophagy inhibitor (bafilomycin A1 [BafA1], prevents maturation of autophagic vacuoles by inhibiting fusion between autophagosomes and lysosomes) were used in the following experiments. DA combined with Z-VAD-FMK did not substantially increase the cell viability of SAS and OECM-1 cells (Figure 1D). Furthermore, cotreatment with DA and BafA1 showed that DA induced a reduction in the percentage of viable cells. However, the viability of SAS and OECM-1 cells increased when BafA1 was included (Figure 1E).

### DA increases cell death as a result of autophagy induction in human oral cancer cell lines

**Figure 2:**
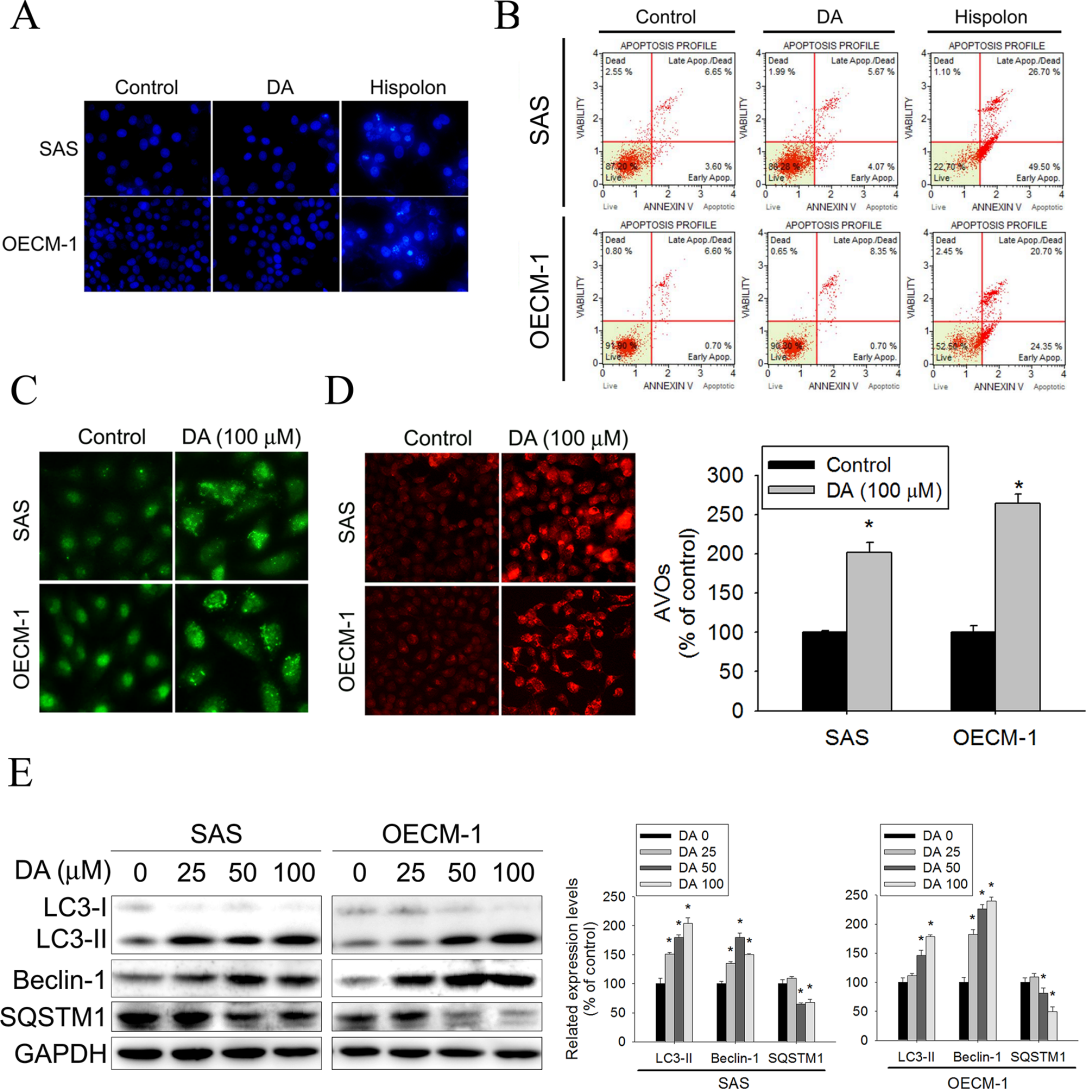
DA increase cell death not due to apoptosis in oral cancer cells **A.** SCC9 and OECM-1 cells seeded onto glass coverslips with treatment with DA (100 μM) or Hispolon (10 μM) for 48 h, followed by immunostaining and an observation of DAPI (blue fluorescence) under fluorescence microscopy. Original magnifications: 200X. **B.** SCC9 and OECM-1 cells treatment with DA (100 μM) or Hispolon (10 μM) for 48 h. Subsequently, these treated cells were double-stained with Annexin-V and PI and then analyzed by flow cytometry. Hispolon treatment conditions as positive control. **C.** SCC9 and OECM-1 cells seeded onto glass coverslips with treatment with DA (100 μM) for 48 h, followed by immunostaining and an observation of LC-3 (green fluorescence) under fluorescence microscopy. Original magnifications: 400X. **D.** SCC9 and OECM-1 cells t treatment with DA (100 μM) for 48 h were stained with acridine orange for AVOs formation. Data shown are representative fluorescence microscopic photographs. Original magnifications: 200X. **E.** SCC9 and OECM-1 cells were treated with DA (100 μM) for 48 h and then subjected to western blotting to study the expression levels of LC3-I/II, SQSTM1 and Beclin-1 with GAPDH acting as an internal control (upper panel). Lower panel, determined expression levels of LC3-II, SQSTM1 and Beclin-1 was subsequently quantified with that of control (DA, 0 μM, 48 h). Results are shown as mean ± SE. *P < 0.05, compared with the control (DA, 0 μM).

The reasons that DA inhibits cell viability were investigated. Autophagy and apoptosis differ in morphological characteristics. Cells undergoing apoptosis exhibit an increase in nuclear chromatin condensation. Autophagy is a type of cell death that occurs in the absence of chromatin condensation but is associated with massive autophagic vacuolization of the cytoplasm. [37] First, we determined whether DA-induced cell death was related to apoptosis. DAPI staining was performed to analyze the changes in nuclear morphology. The results did not show nucleus condensation in both cell types after 48 h of treatment with DA (100 μM) compared with the results of treating both cell types with hispolon (10 μM) (Figure 2A). Treatment with hispolon induces apoptosis (positive control). [38] In addition, annexin-V/PI double staining was performed using flow cytometry. The results showed no significant increase in the percentage of DA (100 μM)-treated SAS and OECM-1 cells exhibiting phosphatidylserine externalization compared with that of hispolon (10 μM)-treated SAS and OECM-1 cells (Figure 2B). Nonapoptotic programmed cell death is ascribed to type II programmed cell death, which is autophagy dependent. Whether autophagy triggers DA-induced human oral cancer cell death was examined. At the beginning of autophagy, the cytosolic form of microtubule-associated protein 1 LC3-I is converted to the phagophore- and autophagosome-bound form of LC3-II. After 48 h of treatment with DA, a substantial change in LC3 puncta formation was observed, indicating the formation of autophagosomes in DA (100 μM)-treated SAS and OECM-1 cells (Figure 2C). Acidic vesicular organelle (AVO) formation (autophagosomes and autolysosomes) is a characteristic feature of autophagy. [39] To detect the development of AVOs, untreated and treated SAS and OECM-1 cells were stained with acridine orange. DA (100 μM) induced AVO formation in SAS and OECM-1 cells (Figure 2D). To determine whether DA induced autophagy, the protein expression of LC3-II, SQSTM1 (p62), and beclin-1 was analyzed. Compared with the control, the protein expression of LC3-II and beclin-1 increased in both cells treated for 48 h with DA (100 μM). However, SQSTM1 expression decreased in DA-treated cells (Figure 2E). These results, combined with the previous results, showed that autophagy was induced by DA.

### Effects of treatment with autophagy enhancers or inhibitors on DA-induced cell death in human oral cancer cell lines

**Figure 3:**
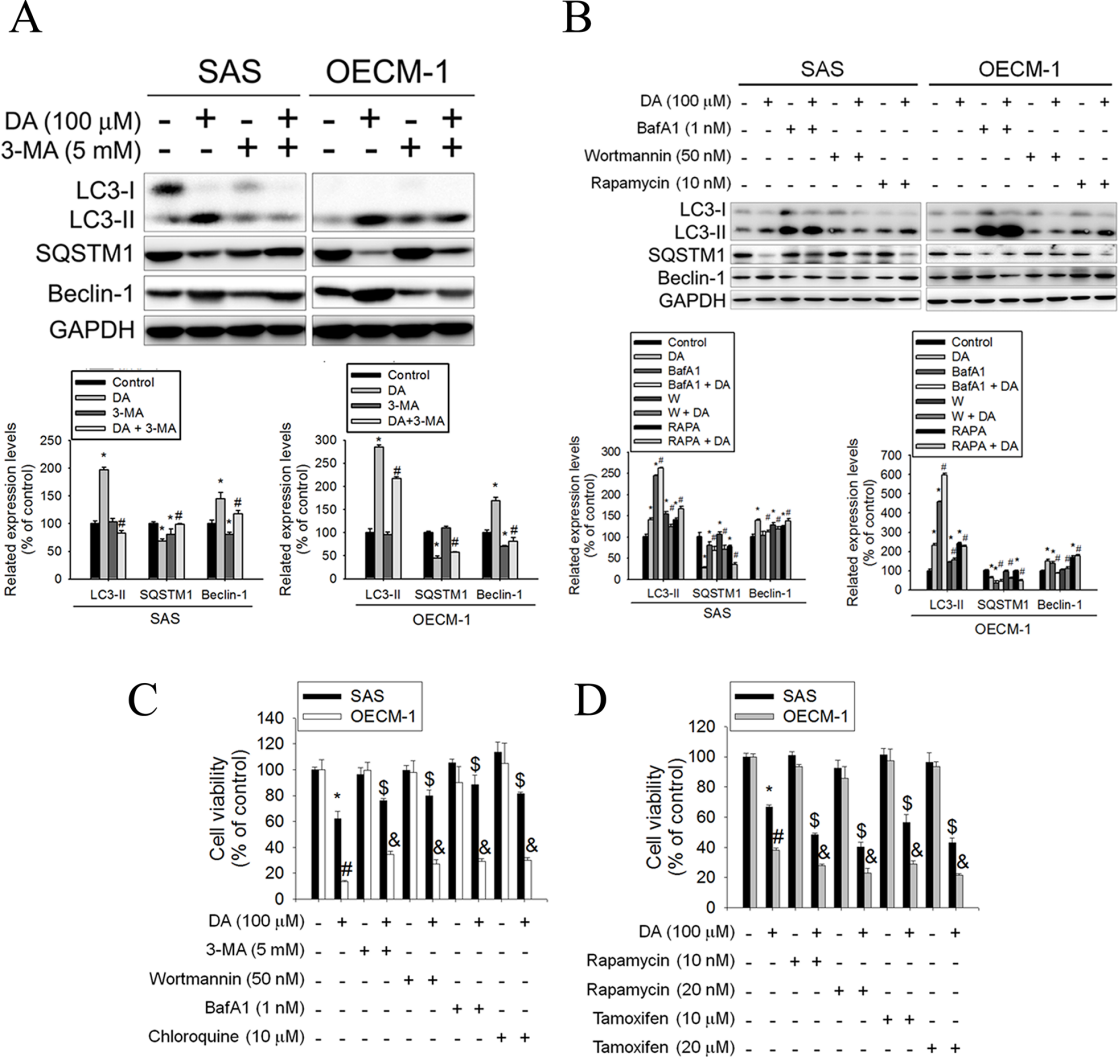
Effects of autophagy enhancers or inhibitors on DA-induced cell death **A.** SAS and OECM-1 cells were treated with DA of 100 μM for 48 h in the presence or absence of autophagy inhibitor 3-MA of 5 mM and then subjected to western blotting for LC3-I/II, SQSTM1 and Beclin-1 with GAPDH acting as an internal control (upper panel). Lower panel, determined expression levels of LC3-II, SQSTM1 and Beclin-1 was subsequently quantified with that of control. Results are shown as mean ± SE. *P < 0.05, compared with the control (no treat). #*P* < 0.05, compared with the control (DA, 100 μM). **B.** SAS and OECM-1 cells were treated with DA of 100 μM for 48 h in the presence or absence of autophagy inhibitor BafA1 (1 nM) and wortmannin (50 nM) or autophagy inducer (rapamycin, 10 nM) and then subjected to western blotting for LC3-I/II, SQSTM1 and Beclin-1 with GAPDH acting as an internal control (upper panel). To determined expression levels of LC3-II, SQSTM1 and Beclin-1 was subsequently quantified with that of control (lower panel). Results are shown as mean ± SE. *P < 0.05, compared with the control (no treat). #*P* < 0.05, compared with the control (DA, 100 μM). **C.** SAS and OECM-1 cells were treated with DA (100 μM) in the presence or absence of autophagy inhibitor 3-MA (5 mM), wortmannin (50 nM), BafA1 (1 nM) or chloroquine (10 μM), as analyzed by MTT assay. Results are shown as mean ± SE. *P < 0.05, #*P* < 0.05, compared with the SAS control (no treat). #*P* < 0.05, compared with the OECM-1 control (no treat). $*P* < 0.05, compared with the SAS treated with DA (100 μM). &*P* < 0.05, compared with the OECM-1 treated with DA (100 μM). **D.** SAS and OECM-1 cells were treated with DA (100 μM) in the presence or absence of autophagy enhancers rapamycin (10 and 20 nM) or tamoxifen (10 and 20 μM) and analyzed by MTT assay. Results are shown as mean ± SE. *P < 0.05, #*P* < 0.05, compared with the SAS control (no treat). #*P* < 0.05, compared with the OECM-1 control (no treat). $*P* < 0.05, compared with the SAS treated with DA (100 μM). &*P* < 0.05, compared with the OECM-1 treated with DA (100 μM).

Autophagy inhibitors (3-methyladenine [3-MA], wortmannin, and BafA1) acting at differing stages were used in the following experiments to elucidate their effect on DA-induced autophagy. 3-MA inhibits autophagy by blocking autophagosome formation via the inhibition of type III Phosphatidylinositol 3-kinases (PI-3K). Wortmannin, PI3K inhibitor, can inhibit autophagic sequestration. BafA1, prevents maturation of autophagic vacuoles by inhibiting fusion between autophagosomes and lysosomes. SAS and OECM-1 cells were pretreated with 3-MA (5 mM), wortmannin (50 nM), or BafA1 (1 nM) for 1 h before treatment with DA; autophagy-related molecules were examined using western blotting. Adding 3-MA combined with DA (100 μM) to SAS and OECM-1 cells resulted in a slight decrease in the levels of LC3-II and beclin-1 (Figure 3A). However, the protein expression of SQSTM1 increased in autophagy inhibitors combined with DA-treated cells. The same results were observed when wortmannin or BafA1 combined with DA was added to SAS and OECM-1 cells (Figure 3B). In addition, rapamycin induces autophagy, because inhibition of mTOR mimics cellular starvation by blocking signals required for cell growth and proliferation. [40] The protein expression of LC3-II and beclin-1 increased in cells treated only with DA, whereas that of SQSTM1 decreased in rapamycin- and DA-treated cells (Figure 3B). The effects of DA combined with autophagy enhancers or inhibitors on cell viability were assessed. SAS and OECM-1 cells were pretreated with 3-MA (5 mM), wortmannin (50 nM), BafA1 (1 nM), or chloroquine (10 μM) for 1 h before treatment with DA (100 μM). Chloroquine inhibits autophagy as it raises the lysosomal pH, which leads to inhibition of both fusion of autophagosome with lysosome and lysosomal protein degradation. [41] After 48 h of treatment, cell viability was analyzed using the MTT assay. DA combined with autophagy inhibitors substantially increased the viability of SAS and OECM-1 cells treated only with DA (Figure 3C). Tamoxifen, an antagonist of the estrogen receptor, stimulates autophagy by increasing the intracellular level of ceramide, which inhibits mTOR activation and stimulates the expression of Atg genes. [42] Furthermore, SAS and OECM-1 cells were pretreated with autophagy enhancers, rapamycin (10 and 20 nM) or tamoxifen (10 and 20 μM) for 1 h before treatment with DA (100 μM) and then analyzed using the MTT assay for 48 h. DA combined with autophagy enhancers reduced the viability of SAS and OECM-1 cells treated only with DA (Figure 3D).

### Autophagy-related gene silencing inhibits DA-induced oral cancer cell death

**Figure 4:**
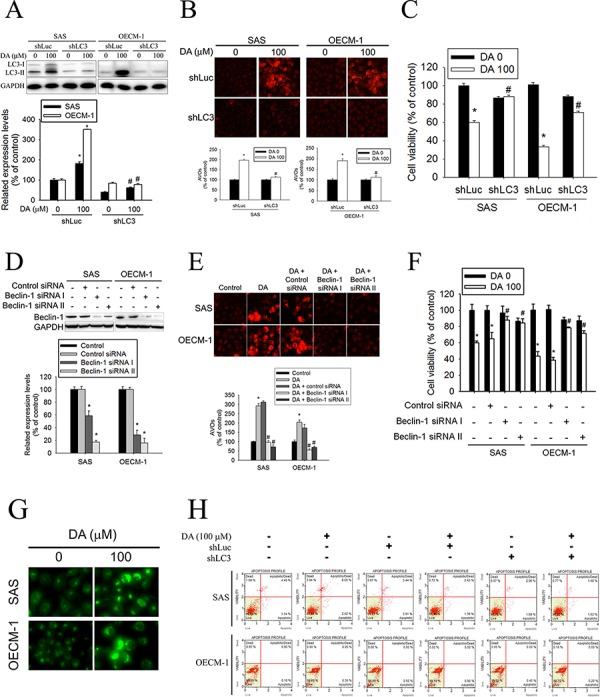
Effect of LC3 silencing and Beclin-1 knockdown on DA-mediated AVOs formation and cell viability **A.** After 100 μM DA treatment for 48 h, total cell lysates from SAS shLuc, OECM-1 shLuc, SAS shLC3 and OECM-1 shLC3 cells (5 × 10^5^ cells/60 mm dish) were analyzed by western blot and detected by LC3 and GAPDH antibodies (upper panel). Lower panel, determined expression levels of LC3-II was subsequently quantified with that of control (DA, 0 μM, 48 h). GAPDH was a loading control. Results are shown as mean ± SE. *P < 0.05, compared with the control (DA, 0 μM). #*P* < 0.05, compared with the DA (100 μM)-treatment shLuc cells. **B.** SAS shLuc, OECM-1 shLuc, SAS shLC3 and OECM-1 shLC3 cells (5 × 10^4^ cells/well of 24-well plate) were treated with or without DA (0 and 100 μM) for 48 h. The cells were stained with acridine orange for AVOs formation and observed under a red filter fluorescence microscope (upper panel). Original magnifications: 200X. Quantitation of the formation of AVOs (lower panel). Results are shown as mean ± SE. *P < 0.05, compared with the DA no treat shLuc cells. #*P* < 0.05, compared with the DA (100 μM)-treatment shLuc cells. **C.** SAS shLuc, OECM-1 shLuc, SAS shLC3 and OECM-1 shLC3 cells (2 × 10^4^ cells/well of 96-well plate) were treated with or without DA (0 and 100 μM) for 48 h and analyzed by MTT assay. Results are shown as mean ± SE. *P < 0.05, compared with the DA no treat shLuc cells. #*P* < 0.05, compared with the DA (100 μM)-treatment shLuc cells. **D.** Cells transfected with 100 nM Control siRNA (Unconjugated), Beclin-1 siRNA I or Beclin-1 siRNA II. Western blot analysis of extracts from SAS and OECM-1 cells, using Beclin-1 antibody confirms silencing of Beclin-1 expression, while the GAPDH antibody is used to control for loading and specificity of Beclin-1 siRNA (upper panel). Lower panel, determined expression levels of Beclin-1 was subsequently quantified with that of control. Results are shown as mean ± SE. *P < 0.05, compared with the control. **E.** After transfected with Control siRNA, Beclin-1 siRNA I or Beclin-1 siRNA II. Cells were treated with or without DA (100 μM) for 48 h. The cells were stained with acridine orange for AVOs formation and observed under a red filter fluorescence microscope (upper panel). Original magnifications: 200×. Quantitation of the formation of AVOs (lower panel). Results are shown as mean ± SE. *P < 0.05, compared with the control. #*P* < 0.05, compared with the DA (100 μM). **F.** After transfected with Control siRNA, Beclin-1 siRNA I or Beclin-1 siRNA II. Cells were treated with or without DA (0 and 100 μM) for 48 h and analyzed by MTT assay. Results are shown as mean ± SE. *P < 0.05, compared with the control (DA, 0 μM, 48 h). #*P* < 0.05, compared with the DA (100 μM). **G.** SAS and OECM-1 cells were treated with DA of 100 μM for 48 h and then stained with MDC visualized at magnification of under a fluorescence microscope. Original magnifications: 200×. **H.** After 100 μM DA treatment for 48 h, SAS shLuc, OECM-1 shLuc, SAS shLC3 and OECM-1 shLC3 cells were double-stained with Annexin-V and PI and then analyzed by flow cytometry. Results are shown as mean ± SE. *P < 0.05, compared with the DA no treat shLuc cells. #*P* < 0.05, compared with the DA (100 μM)-treatment shLuc cells.

To clarify the role of autophagy in DA-mediated oral cancer cell death, an LC3 silencing experiment was conducted using a VZV-G pseudotyped lentivirus–shRNA system. The responses of SAS shLuc cells and OECM-1 shLuc cells were similar to those of parental SAS and OECM-1 cells after treatment with DA. In SAS shLC3 cells and OECM-1 shLC3 cells, DA-induced LC3-II conversion was blocked (Figure 4A). Compared with AVO formation in SAS shLuc cells and OECM-1 shLuc cells, DA-induced AVO formation was blunted in SAS shLC3 cells and OECM-1 shLC3 cells (Figure 4B). The results suggested that LC3 silencing abolishes autophagy in SAS and OECM-1 cells treated with DA. Furthermore, the cytotoxic effect of DA (100 μM) was significantly decreased in SAS shLC3 cells and OECM-1 shLC3 cells (Figure 4C). Investigators have demonstrated that beclin-1^−/−^ mice die early in embryogenesis and beclin-1^−/+^ mice have a high incidence of spontaneous tumors. Stem cells from null mice exhibited an altered autophagic response even though responses to apoptosis appeared normal. [43] Extracts from SAS and OECM-1 cells transfected with control siRNA, beclin-1 siRNA I, or beclin-1 siRNA II were subjected to western blot analysis. Beclin-1 antibody confirms silencing of beclin-1 expression (Figure 4D). DA-induced AVO formation decreased in SAS-transfected beclin-1 siRNA cells compared with DA-treated SAS cells (Figure 4E). The same results were observed in OECM-1 cells. In addition, DA-mediated cell viability was significantly increased in beclin-1 knockdown cell lines (Figure 4F). MDC, a specific autophagolysosome marker to analyze at the molecular level the machinery involved in the autophagic process, was used to further investigate the formation of autophagosome. The results showed that the number of MDC-labeled vesicles in DA-treated cells was increased (Figure 4G). To clarify the DA-induced cell death mode in OSCC, the effect of LC3 knockdown on DA-induced cell death was detected by Annexin-V/PI double staining. The results showed no significant increase in Annexin-V/PI double staining in the DA (100 μM)-treated SAS shLC3 cells and OECM-1 shLC3 cells (Figure 4H).

### Effects of inhibiting p53 expression on DA-induced autophagy

**Figure 5:**
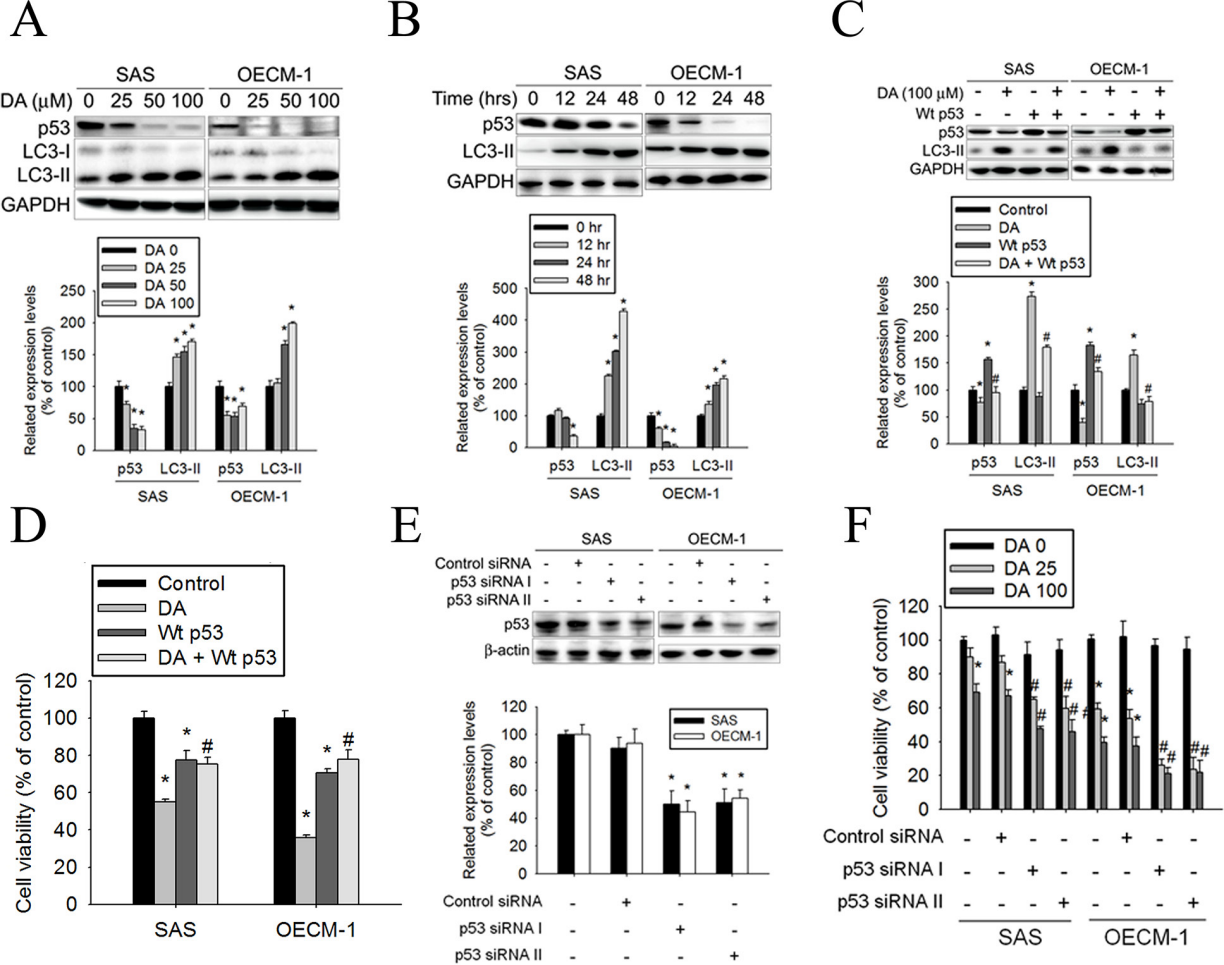
Effects of p53 expression on DA-induced autophagy **A.** SAS and OECM-1 cells were treated with DA (0–100 μM) for 48 h and then subjected to western blotting for p53 and LC3-I/II with GAPDH acting as an internal control (upper panel). Lower panel, determined expression levels of p53 and LC3-II was subsequently quantified with that of control. Results are shown as mean ± SE. *P < 0.05, compared with the control (no treat). **B.** SAS and OECM-1 cells cultured in presence of various concentrations of DA (100 μM) for 12, 24 and 48 h and then subjected to western blotting for p53 and LC3-I/II with GAPDH acting as an internal control (upper panel). Lower panel, determined expression levels of p53 and LC3-II was subsequently quantified with that of control. Results are shown as mean ± SE. *P < 0.05, compared with the control (no treat). **C.** After transfected, cells were treated with or without DA (0 and 100 μM) for 48 h and then analyzed by western blot and detected by p53 and LC3-II antibodies (upper panel). Lower panel, determined expression levels of p53 and LC3-II were subsequently quantified with that of control (DA, 0 μM, 48 h). GAPDH was a loading control. Results are shown as mean ± SE. *P < 0.05, compared with the control (DA, 0 μM). #*P* < 0.05, compared with the DA (100 μM)-treatment cells. **D.** After transfected, cells were treated with or without DA (0 and 100 μM) for 48 h and analyzed by MTT assay. Results are shown as mean ± SE. *P < 0.05, compared with the control (DA, 0 μM). #*P* < 0.05, compared with the DA (100 μM)-treatment cells. **E.** Cells transfected with 100 nM Control siRNA (Unconjugated), p53 siRNA I or p53 siRNA II. Western blot analysis of extracts from SAS and OECM-1 cells, using p53 antibody confirms silencing of p53 expression, while the GAPDH antibody is used to control for loading and specificity of p53 siRNA (upper panel). Lower panel, determined expression levels of p53 was subsequently quantified with that of control. Results are shown as mean ± SE. *P < 0.05, compared with the control. **F.** After transfected with Control siRNA, p53 siRNA I or p53 siRNA II. Cells were treated with or without DA (25 or 100 μM) for 48 h and analyzed by MTT assay. Results are shown as mean ± SE. *P < 0.05, compared with the control (DA, 0 μM). #*P* < 0.05, compared with the DA (100 μM)-treatment cells.

A previous study showed that inhibition of p53 led to autophagy in enucleated cells, and cytoplasmic, not nuclear, p53 repressed the enhanced autophagy of p53^−/−^ cells. [44] Furthermore, inhibition of p53 degradation prevented the activation of autophagy. These results provide evidence of a key signaling pathway that links autophagy to the cancer-associated dysregulation of p53. [44] To clarify the associated mechanisms of DA-induced autophagy in oral cancer cells, the p53 expression levels in DA-treated oral cancer cells were detected using western blotting. The results showed that p53 expression decreased in DA-treated oral cancer cells along with DA-stimulated LC3-II induction (Figure 5A). In addition, DA (100 μM) suppressed p53 expression and induced LC3-II conversion in a time-dependent manner (Figure 5B). For further confirmation of the connection between p53 expression and autophagy induction in DA-treated oral cancer cells, SAS and OECM-1 cells were transfected with Wt-p53 and subsequently treated with DA. DA-induced LC3-II expression decreased in Wt-p53-transfected oral cancer cells compared with that in the cells treated only with DA (Figure 5C). In addition, the DA-mediated cell viability of oral cancer cells after p53 overexpression by transfected Wt-p53 was measured. It was noted that p53 overexpression reduced the cell-killing effect of DA (Figure 5D). Conversely, when p53 expression was knocked down by p53 siRNA, viability was significantly inhibited in DA-treated cells (Figure 5E and 5F). These data suggested that p53 plays a vital role in DA-induced autophagy regulation.

### DA-stimulated autophagy promotes the AMPK pathway and influence mTOR signaling

**Figure 6:**
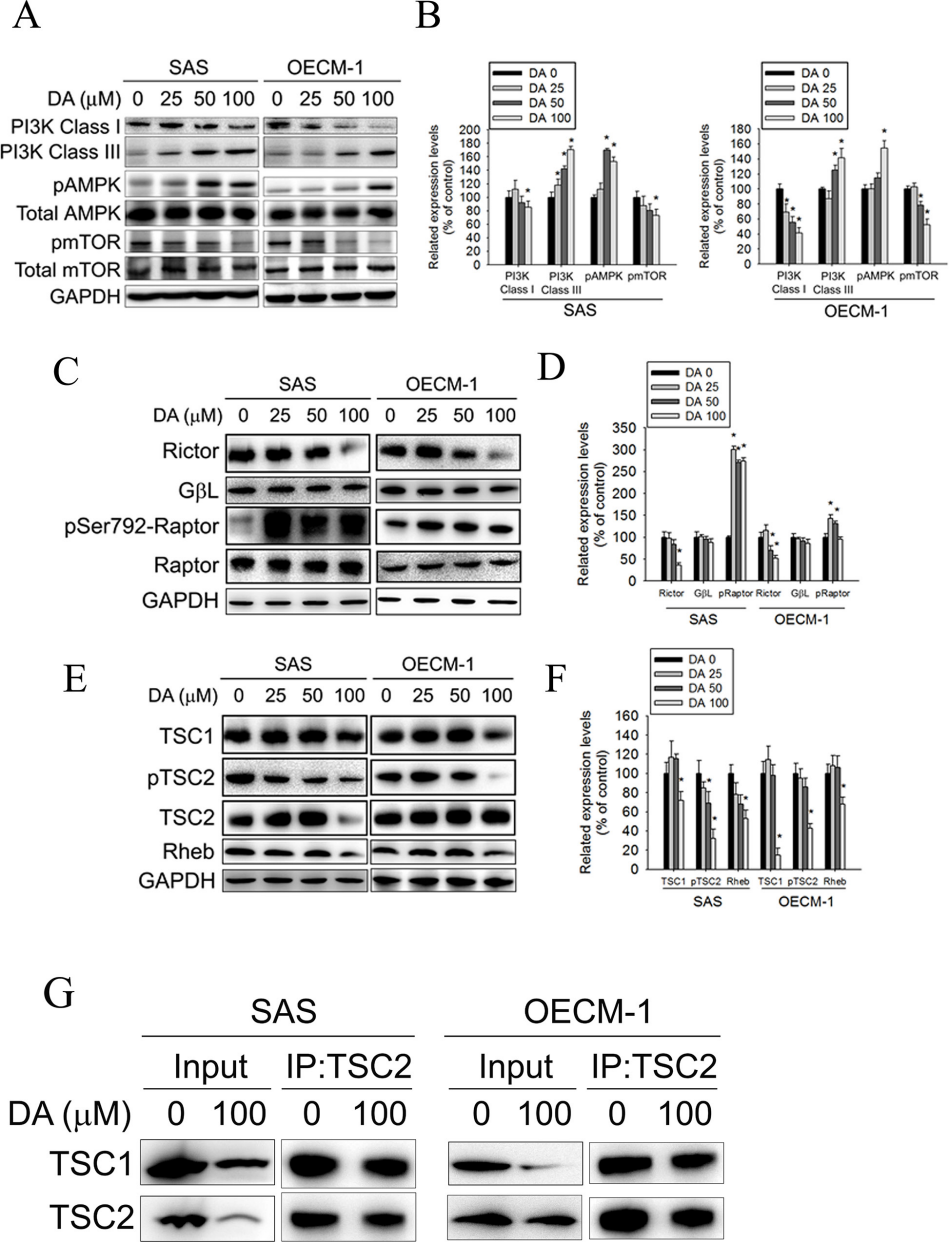
Effect of PI3K, AMPK, mTOR complex and TSC expression on DA-induced autophagy **A.** SAS and OECM-1 cells were treated with DA (0–100 μM) for 48 h and then subjected to western blotting for Class I PI3K, Class III PI3K, AMPK and mTOR with GAPDH acting as an internal control. **B.** Determined expression levels of Class I PI3K, Class III PI3K, phospho-AMPK and phospho-mTOR was subsequently quantified with that of control. Results are shown as mean ± SE. *P < 0.05, compared with the control (no treat). **C.** SAS and OECM-1 cells were treated with DA (0–100 μM) for 48 h and then subjected to western blotting for Rictor, GβL and Raptor with GAPDH acting as an internal control. **D.** Determined expression levels of Rictor, GβL and phospho-Raptor was subsequently quantified with that of control. Results are shown as mean ± SE. *P < 0.05, compared with the control (no treat). **E.** SAS and OECM-1 cells were treated with DA (0–100 μM) for 48 h and then subjected to western blotting for TSC1, TSC2 and Rheb with GAPDH acting as an internal control. **F.** Determined expression levels of TSC1, phospho-TSC2 and Rheb was subsequently quantified with that of control. Results are shown as mean ± SE. *P < 0.05, compared with the control (no treat). **G.** SAS and OECM-1 cells were treated with DA (0–100 μM) for 48 h. TSC-2 was immunoprecipitated from SAS and OECM-1 cells treated with DA (0–100 μM) for 24 h and then subjected to western blotting for TSC1 and TSC2.

**Figure 7:**
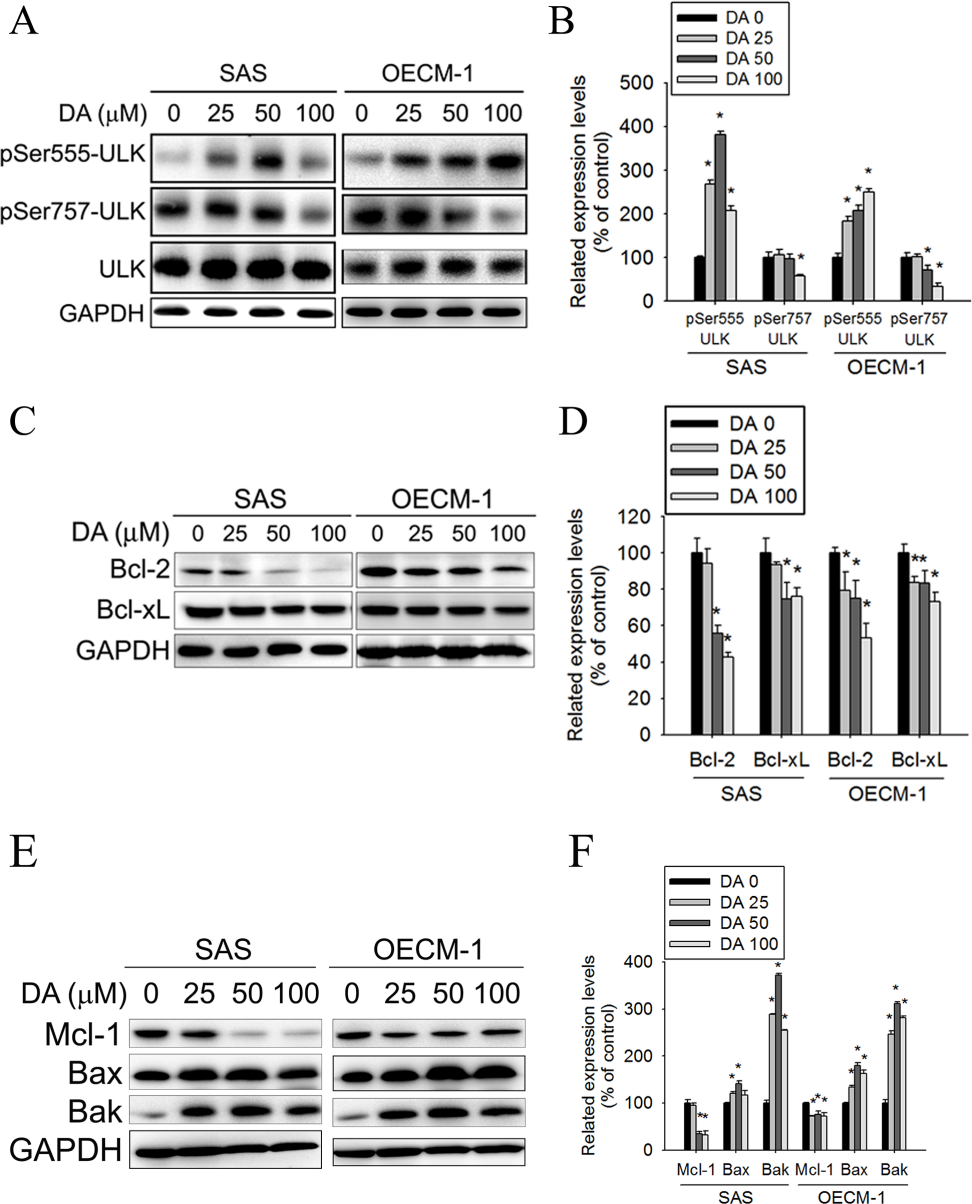
Effect of ULK, Bcl-2 and Bcl-xL on DA-induced autophagy **A.** SAS and OECM-1 cells were treated with DA (0–100 μM) for 48 h and then subjected to western blotting for ULK with GAPDH acting as an internal control. **B.** Determined expression levels of phospho-Ser555-ULK and phospho-Ser757-ULK was subsequently quantified with that of control. Results are shown as mean ± SE. *P < 0.05, compared with the control (no treat). **C.** SAS and OECM-1 cells were treated with DA (0–100 μM) for 48 h and then subjected to western blotting for Bcl-2 and Bcl-xL with GAPDH acting as an internal control. **D.** Determined expression levels of Bcl-2 and Bcl-xL was subsequently quantified with that of control. **E.** SAS and OECM-1 cells were treated with DA (0–100 μM) for 48 h and then subjected to western blotting for Mcl-1, Bax and Bak with GAPDH acting as an internal control. **F.** Determined expression levels of Mcl-1, Bax and Bak was subsequently quantified with that of control. Results are shown as mean ± SE. *P < 0.05, compared with the control (no treatment).

The kinase mTOR is a critical regulator of autophagy induction, with activated mTOR (Akt and mitogen-activated protein kinase [MAPK] signaling) suppressing autophagy and negative regulation of mTOR (AMPK and p53 signaling) promoting it. Class I PtdIns 3-kinase generates PtdIns (3,4) P2 and PtdIns (3,4,5) P3, which bind to the pleckstrin homology domain of Akt and its activator 3-phosphoinositide-dependent protein kinase-1. [45] When the Akt signaling pathway is activated, autophagic degradation is reduced. The Class III PI3K complex, which contains beclin-1, p150, and Atg14-like protein or the ultraviolet-irradiation-resistance-associated gene, is required for the induction of autophagy. The Class III PI3K is a critical regulator of autophagy [46]. To investigate the molecular mechanisms of DA-induced autophagy, Class I PI3K, Class III PI3K, and AMPK expression levels and mTOR kinase activity, as measured by its phosphorylation, were examined using DA. In DA-treated oral cancer cells, Class I PI3K expression decreased (Figure 6A and 6B), whereas Class III PI3K expression increased. In addition, DA significantly induced AMPK phosphorylation and inhibited mTOR phosphorylation compared with the vehicle control. Inhibition of mammalian target of rapamycin complex 1 (mTORC1) correlates with increased autophagy [47]. To further investigate the mechanism of DA-induced autophagy in human oral cancer cell lines, the expression levels of the mTOR complex-related proteins, Raptor and TSC were examined. The results showed that DA treatment resulted in the activation of Raptor at Ser792. By contrast, the level of Rictor, TSC1, Rheb and phosphorylated TSC2 were decreased in both cell lines (Figure 6C–6F). According to a previous study showing that TSC1/2 protein complex negatively regulates the mTORC1 as master regulator of protein synthesis, cell growth and autophagy [48, 49], we investigated the association between TSC-1 and TSC-2 in DA treatment cell lines. As shown in Figure 6G, the protein–protein binding between TSC-1 and TSC-2 was analyzed by immunoprecipitating to indicate that DA significantly disrupts the binding between TSC-1 and TSC-2. ULK induces autophagy by phosphorylating beclin-1 and activating VPS34 lipid kinase [50]. The possibility of direct control of ULK via phosphorylation by AMPK, mTORC1 may negatively regulate ULK by phosphorylation [47]. In this study, DA treatment resulted in the activation of ULK at Ser555. By contrast, the level of phosphorylated ULK at Ser757 was decreased in both cell lines (Figure 7A and 7B). Previous studies have reported that Bcl-2 and Bcl-xL inhibited beclin-1-mediated autophagy by binding to beclin-1. In addition, beclin-1, Bcl-2, and Bcl-xL can cooperate with Atg5 or Ca^2+^ to regulate both autophagy and apoptosis. Thus, Bcl-2 and Bcl-xL play crucial roles in the cross talk between autophagy and apoptosis [51]. In this study, Bcl-2 and Bcl-xL expression decreased in DA-treated oral cancer cells (Figure 7C and 7D). The expression level of Mcl-1 has been suggested to regulate autophagic flux. Specifically, deletion of Mcl-1 in cortical neurons of transgenic mice has been found to activate a robust autophagic response. [52] In addition, when Bax and Bak were present, inhibiting the prosurvival Bcl-2 family members stimulated autophagy. [53, 54] In this study, treatment with DA inhibited Mcl-1 expression; however, the Bax and Bak expression levels increased in DA-treated cells (Figure 7E and 7F). These results demonstrate that DA-induced autophagy through promotes the AMPK pathway and influence the expression of mTOR complex-related proteins and ULK. In addition, DA induces beclin-1-mediated autophagy by inhibiting Bcl-2, Bcl-xL and Mcl-1 expression.

### DA induces cell autophagy by inhibiting Akt and p38 phosphorylation and enhancing JNK1/2 activation

**Figure 8:**
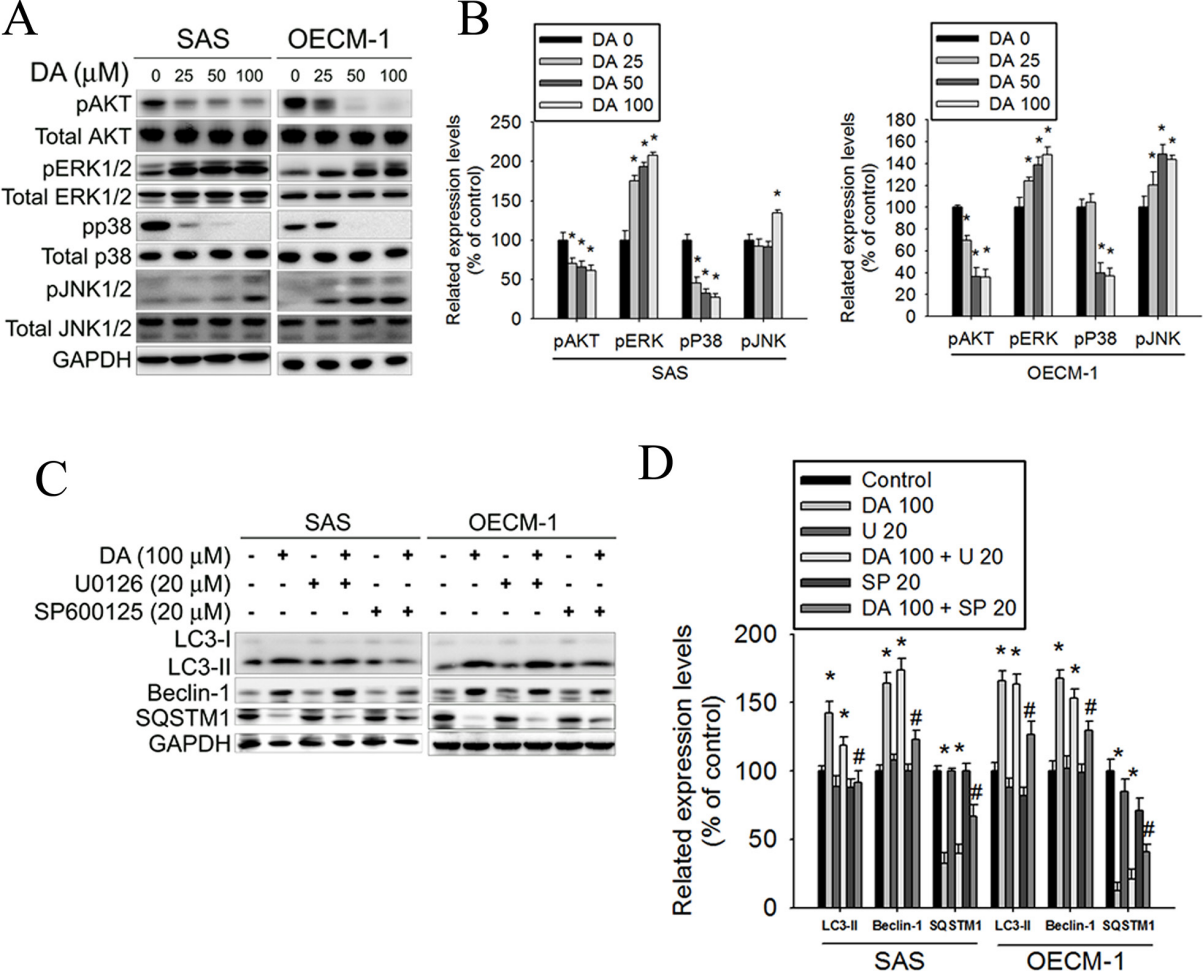
Effect of Akt and MAPK pathway on DA induces cell autophagy processes **A.** SAS and OECM-1 cells were treated with DA (0–100 μM) for 48 h and then subjected to western blotting for Akt, ERK1/2, p38 and JNK1/2 with GAPDH acting as an internal control. **B.** Determined expression levels of phospho-Akt, phospho-p38, phospho-ERK1/2 and phospho-JNK1/2 was subsequently quantified with that of control. Results are shown as mean ± SE. *P < 0.05, compared with the control (no treat). **C.** SAS and OECM-1 cells were treated with DA of 100 μM for 48 h in the presence or absence of specific inhibitors of ERK1/2 (U0126) and JNK1/2 (SP600125) of 20 μM and then subjected to western blotting for LC3-I/II, SQSTM1 and Beclin-1 with GAPDH acting as an internal control. **D.** Determined expression levels of LC3-II, SQSTM1 and Beclin-1 was subsequently quantified with that of control. Results are shown as mean ± SE. *P < 0.05, compared with the control (no treat). #*P* < 0.05, compared with the control (DA, 100 μM).

MAPK signaling plays a critical role in the outcome of and sensitivity to anticancer therapies. Apoptosis and autophagy can be induced by extracellular stimuli such as treatment with chemotherapeutic agents, resulting in different cell responses to these drugs. [55] To further clarify the association between DA-induced autophagy and MAPK signaling, the activation of MAPK was investigated in DA-treated oral cancer cells. In this study, treatment with DA inhibited Akt and p38 phosphorylation; however, the ERK1/2 and JNK phosphorylation expression levels increased in DA-treated cells (Figure 8A and 8B). According to previous studies, we investigated the effects of DA on specific inhibitors of ERK1/2 (U0126) and JNK1/2 (SP600125) in SAS and OECM-1 cells. The data showed that combined treatment with the JNK1/2 inhibitor and DA at 100 μM further reduced LC3-II and beclin-1 expression. The SQSTM1 expression levels were increased by combined treatment with the JNK1/2 inhibitor and DA at 100 μM (Figure 8C and 8D). Conversely, the results showed that combined treatment with DA and ERK1/2-specific inhibitors did not influence the LC3-II, beclin-1, and SQSTM1 expression levels (Figure 8C and 8D). Therefore, the inhibition of Akt, p38 phosphorylation, and enhanced JNK1/2 signaling pathways may result in DA-induced autophagy.

### Significant antiproliferative effects of DA in an SAS orthotopic graft model

**Figure 9:**
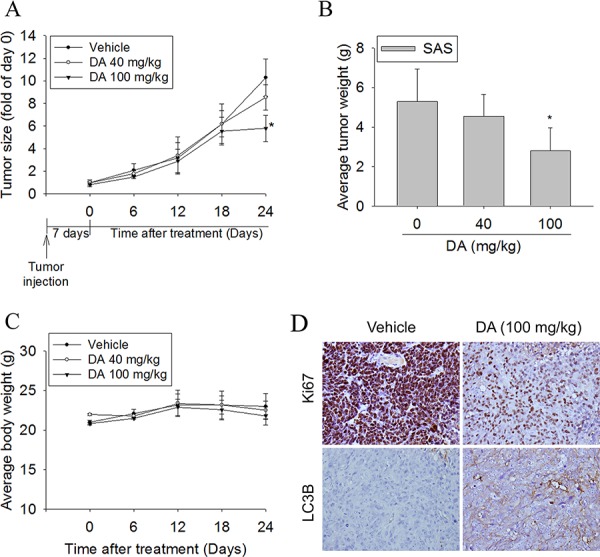
DA suppressed tumor growth SAS cells *in vivo* SAS cells were injected into the right flank of 6-week-old male BALB/c nude mice. After injection of SAS cells, nude mice were treated with either vehicle or DA (40 or 100 mg/kg) for 24 days. **A.** The growth of the xenograft tumors was referred to the measurement of the long and short dimensions of the tumors, and the calculation of the tumor size were described in the “Materials and methods” section. **B.** Tumor weight changes of the mice during 24 days of treatment. **C.** Body weight changes of the mice during 24 days of treatment. *P < 0.05, compared to the vehicle. **D.** Tumor tissues were examined by H&E staining, and immunohistochemical staining with an anti-LC3 antibody. A proliferation index was determined based on Ki67 immunostaining. Original magnifications: 400×.

To test the effect of DA on tumor growth, the *in vivo* antitumor effect of DA was evaluated. Tumor volumes were determined by caliper measurements for every 6 days. The control group of animals transplanting SAS cancer cells showed a progressive increase in their tumor volumes. In DA-treated mice receiving 100 but not 40 mg/kg, the mean tumor volume on day 24 was significantly inhibited compared to vehicle-treated (Figure 9A). As illustrated in Figure 9B, the mean tumor weight in DA-treated mice was smaller compared to that of mice injected with only vehicle. No significant difference in body weight was detected among these groups (Figure 9C). In IHC analysis for cell proliferation, Ki67-positive tumor cells were significantly reduced after treatment with DA compared to control mice. In addition, the expression of LC3 in tumor specimens was increased in the DA-treated group compared to the control group (Figure 9D). These results showed that animals treated with DA show anti-tumor growth as compared with control animals.

## DISCUSSION

Autophagy is a major intracellular degradation mechanism that causes programmed type II cell death under specific conditions. [5–7] Autophagy is a multifaceted process, and alterations in autophagic signaling pathways are frequently observed in cancer. Previous studies have reported that different ROS have different effects on autophagy. Superoxides induce autophagy, [56] and NOs impair autophagosome synthesis. Overexpression of nNOS, iNOS, or eNOS inhibits autophagic flux primarily through the JNK1–Bcl-2 pathway. Conversely, NOS inhibition enhances the clearance of autophagic substrates. [28] These results suggest that autophagy induction may trigger the inhibition of NOS expression. Our data suggest that distinct mechanisms related to DA induce autophagy. First, DA inhibits p53 expression, leading to autophagy in oral cancer cells. In this study, DA suppressed p53 expression in a dose- and time-dependent manner (Figure 5A and 5B). In addition, p53 overexpression reduced LC3-II expression (Figure 5C) and the percentage of DA-induced cell death (Figure 5D). Conversely, when p53 expression was reduced by transfecting cells with specific p53 siRNA, DA-induced autophagy cell death increased (Figure 5F). Second, Akt and mTOR phosphorylation decreased in a manner that was related to treatment with DA (Figures 5A and 6A). Previous studies have reported that AMPK and mTOR regulate autophagy through direct Ulk1 phosphorylation, and activated AMPK promotes the inhibition of mTOR kinase. [57] In this study, AMPK phosphorylation increased in DA-treated cells (Figure 5A). In addition, DA specifically inhibited Class I PI3K expression and increased Class III PI3K expression. These results are consistent with those of the previous studies. [58] Wortmannin and 3-MA are inhibitors of PI3K that blocks autophagy at an early stage; 3-MA inhibits autophagy by inhibiting Class III PI3K expression and, thus, blocking autophagosome formation. [59, 60] LC3-II, beclin-1, and SQSTM1 were affected by treatment with 3-MA or wortmannin. In addition, DA-induced cell death decreased under 3-MA or wortmannin treatment conditions (Figure 3). In the presence of BafA1, accumulation of LC3-II-positive autophagosomes would be evidence of efficient autophagic flux, while failure of LC3-II protein to increase in the presence of BafA1, would indicate a defect or delay earlier in the process, prior to degradation at the autolysosome. LC3-II accumulation is increased in the presence of BafA1. [61, 62] These results reveal that DA induces autophagy cell death in oral cancer cells by inhibiting the PI3K/Akt/mTOR pathway. Third, DA induces cell autophagy by reducing Akt and p38 phosphorylation and by inducing JNK1/2 activation. Previous studies have shown that SAHA-induced autophagy in glioblastoma stem cells depends the inhibition of mTOR through the inhibition of Akt activation. [63] Wang et al. determined that active Akt can inhibit autophagy through mTOR-independent mechanisms and Akt-mediated regulation of autophagy through beclin-1 phosphorylation. [64] Autophagy deficiency increases the activation of p38 [65]. The p38 MAPK has a dual role in the regulation of autophagy, as both a positive and negative regulator. [55] Our data show that DA inhibits Akt and p38 phosphorylation. The results are consistent with those of previous studies. [64, 66] Furthermore, our data suggest that DA induces autophagy by activating JNK1/2 but not ERK1/2 (Figure 8C and 8D). These results are consistent with those of previous studies [67, 68] and indicate that DA may be involved in other cellular signaling responses. Fourth, DA inhibits Bcl-2, Bcl-xL and Mcl-1 expression in oral cancer cells. Bcl-2 and Bcl-xL inhibit autophagy by binding to beclin-1. The inhibition of Mcl-1 is hypothesized to induce autophagic cell death. In this study, Bcl-2, Bcl-xL and Mcl-1 expression levels decreased in DA-treated oral cancer cells. Furthermore, DA-induced autophagy enhanced JNK1/2 signaling pathways (Figure 8C and 8D). Activation of JNK1, but not JNK2, has been recently shown to mediate starvation-induced autophagy in mammalian cells by phosphorylating Bcl-2. [69] These results show that one of the distinct mechanisms associated with DA-induced autophagy may inhibit Bcl-2 and Bcl-xL expression or influence Bcl-2 phosphorylation by increasing JNK activation, which reduces Bcl-2–beclin 1 bind. However, additional experimental studies are required to confirm this finding. Finally, an administration of DA effectively suppressed the tumor formation in the xenograft model *in vivo* (Figure 9). The mean tumor volume in DA treated mice was smaller compared to control, however, is somewhat at odds with the *in vitro* data (Figure 1).

In conclusion, our results reveal that DA inhibits cell viability and induces autophagy in oral cancer cells, suggesting that DA, an iNOS inhibitor, is an attractive candidate for tumor therapies. DA could serve as a new and potential chemopreventive agent for treating human oral cancer.

## EXPERIMENTAL PROCEDURES

### Cell lines

The SAS and OECM-1 human oral cancer cell lines were purchased from ATCC (ATCC: American Type Culture Collection, Manassas, VA, USA). SAS cells were cultured in Dulbecco's modified Eagle's medium-F12 supplemented with 10% fetal bovine serum (FBS), 1% penicillin/streptomycin, 1.5 g/L sodium bicarbonate, 25 mM HEPES and 1 mM sodium pyruvate (Sigma, St. Louis, Mo, USA). OECM-1 cells were cultured in Roswell Park Memorial Institute (RPMI) 1640 medium supplemented with 10% FBS, 1% penicillin/streptomycin, 1.5 g/L sodium bicarbonate, 25 mM HEPES and 1 mM sodium pyruvate (Sigma, St. Louis, Mo, USA). The cells culture weas maintained at 37°C in a humidified atmosphere of 5% CO_2_.

### Antibodies and other reagents

Dehydroandrographolide (DA) of ≥98% (HPLC) purity was purchased from ChemFaces. Stock solution of DA was made at 25, 50 and 100 mM concentration in dimethyl sulfoxide (DMSO) and stored at −20°C. The final concentration of DMSO for all treatments was less than 0.2%. Other chemicals and rapamycin were obtained from Sigma Chemical Co. Caspases inhibitor (Z-VAD-FMK), autophagy inhibitors (3-methyladenine, bafilomycin A1, wortmannin, choloquine) and autophagy inducer (tamoxifen) were obtained from LC Laborstories, St. Woburn, MA, USA. Hispolon, ≥98% (HPLC) powder, ERK1/2 inhibitor (U0126) and JNK1/2 inhibitor (SP600125) were purchased from Santa Cruz Biotechnology (Santa Cruz, CA). The Muse Annexin V & Dead Cell Assay Kit (MCH100105) was obtained from Meck Millipore. Control siRNA, p53 siRNA, beclin-1 siRNA and antibodies were obtained from Cell Signaling. Wild-type p53 expression plasmid was obtained from Sino Biological, Inc (pCMV3-GFPSpark-TP53).

### Cell cytotoxicity

As previously described [2]. Briefly, 0.5 × 10^4^ cells were cultured in 96-well plates and stimulated with different concentrations of DA. After 24, 48 or 72 h incubation. The medium was removed, 0.5 mg/ml MTT ([3-(4,5-Dimethyl-2-Thiazolyl)-2,5-Diphenyl Tetrazolium Bromide; Thiazolyl Blue Tetrazolium Bromide]) (Affymetrix, Santa Clara, California, USA.) was added to each well and incubated for further 4 h. The viable cell number was directly proportional to the production of formazan, reflected by the color intensity measured at 595 nm, following the solubilization with isopropanol.

### Colony formation

SAS and OECM-1 cell line was seeded at a concentration of 1 × 10^4^ cells per well in 6-well cell culture plates in appropriate media. After 24 h, media was replaced with fresh media containing either DA at 25, 50 and 100 μM. Medium with compound being changes every 3 days. After 2 weeks, colonies were stained with 0.3% crystal violet solution.

### DAPI (2-(4-Amidinophenyl)-6-indolecarbamidine dihydrochloride) staining

5 × 10^4^ cells were grown on 24-well cell culture dish overnight. After being subjected to indicate treatment, cells were fixed with 4% paraformaldehyde for 20 minutes. Extensive PBS washing was conducted between each reaction to remove any residual solvent. Cells were subjected to DAPI staining for 10 minutes and then observed under fluorescence microscopy equipped with filters for UV.

### Annexin V/PI double staining

An Muse Annexin V & Dead Cell Assay Kit was used to quantify cell numbers in different stages of cell death. Briefly, Prepare cell samples (100 μl) and incubation with Muse Annexin V & Dead Cell reagent (100 μl) for 20 min at room temperature. Percentage of live, early apoptotic, late apoptotic, total apoptotic, and dead cells. The Muse Annexin V & Dead Cell Assay is for use with the Muse™ Cell Analyzer.

### *In situ* immunofluorescence assay

Cell were seeded into 6-well dish at a density of 4 × 10^5^ cells per dish. After DA incubation, cells were fixed with 4% paraformaldehyde for 20 min and then incubated with 0.5% Triton X-100 for 10 min. PBS washing was conducted between each reaction to remove any residual solvent. Afterwards, fixed cells were incubated with 4% BSA at room temperature for 2 h and then with the appropriate primary antibodies at 4°C for overnight. After overnight incubation, cells were washed and then incubated with Alexa Fluor 488-conjugated affinipure goat anti-rabbit IgG secondary antibody (Jackson Immuno Research, West Grove, PA, USA) with light protection for 1 h. At the end of incubation, cells were observed under fluorescence microscopy equipped with filters for UV and Blue 488 nm.

### Detection of acidic vesicular organelle (AVO) formation

DA-treated cells were washed with PBS, followed by staining with 1 μg/ml acridine orange for 30 min, 37°C. Afterwards, cells were washed with PBS and then observed under fluorescence microscopy equipped with filters for 488 nm. For quantification of AVOs, acridine orange-stained cells were harvested, washed twice with PBS, resuspended in PBS containing 5% FBS and then analyzed by flow cytometry.

### Western blot analysis

Cell lysates were separated in a polyacrylamide gel and transferred onto a PVDF membrane. The blot was subsequently incubated with 5% non-fat milk in PBS for 1 h and probed with a corresponding antibody against a specific protein for 37°C at 2 h or overnight at 4°C, and then with an appropriate peroxidase conjugated secondary antibody for 1 h. Signal was developed by ECL detection system and relative photographic density was quantitated by a gel documentation and analysis system.

### VZV-G pseudotyped lentivirus-shRNA production system

The RNAi reagents were obtained from the National RNAi Core Facility located at the Institute of Molecular Biology/Genomic Research Center, Academia Sinica. Individual clones are identified by their unique TRC number: shLuc TRCN0000072246 for vector control targeted to luciferase, shLC3 (91) TRCN0000243391 (responding sequence: AGC GAG TTG GTC AAG ATC ATC) targeted to LC3.

### Lentivirus-shRNA infection of cells

5 × 10^5^ cells were cultured onto 60 mm plates. After 16 h of culture, cells were infected with recombinant lentivirus vectors. The next day, the medium was removed and the cells were selected by 2 μg/ml puromycin for 48 h.

### Cell transfection

Cell were seeded into 12-well dish at a density of 2 × 10^5^ cells per dish. Mix 100 μl of serum-free medium and 2 μl of Transfection Reagent by pipetting at room temperature for 5 min. Add 6 μl of 10 μM stock siRNA to the microfuge tube to yield a final concentration of 100 nM or 2 ug wild-type p53 plasmid, incubate for 5 min at room temperature and then add in 12-well. After 24 h, replace the medium with fresh medium. Examine specific siRNA-transfected cells using western blot analysis to determine transfection efficiency.

### Detection of autophagic vacuoles development

Autofluorescent drug monodansylcadaverine (MDC), a specific autophagolysosome marker to analyze at the molecular level the machinery involved in the autophagic process was utilized. In brief, the treated cells were washed with PBS twice, stained with 1 mg/ml MDC (Sigma), and diluted in PBS containing 5% FBS for 15 min. After staining, the cells were washed with PBS, covered with PBS containing 5% FBS, and observed under a green-filter fluorescence microscope.

### *In vivo* anti-tumor growth effects on xenograft transplantation

For experimental xenograft growth inhibition study, 5–6 week male BALB/c nude mice (18–22 g) (National Taiwan University Animal Center, Taiwan) were used. SAS cells (2 × 10^6^ per mouse) were resuspended in 200 μl of sterile PBS and injected s.c. into the right flank of the mouse. Mice were randomized into two groups (5 mice/group). All animals were housed with a regular 12-h light/12-h dark cycle and water ad libitum access to standard rodent chow diet (LaboratoryRodent Diet 5001, LabDiet, St. Louis, MO), and kept in a pathogen-free environment at the Laboratory Animal Unit (temperature 22°C, humidity 30~70%, 5 mice/cage). Seven days after tumor cell injection, the mice were orally fed DA (40 or 100 mg/kg) or vehicle three times per week. The control group received an equal volume of 0.5% carboxymethyl cellulose vehicle. Tumor volumes were determined from caliper measurements obtained every six days. At the end of the experiment, mice were animals were sacrificed and primary tumors were removed for further analysis. The primary tumors were separated from the surrounding muscles and dermis, and then weighed. The tumor volume was calculated by the following formula: 0.5 × length × width^2^. Mean weight of mice at initiation of study and termination of study did not differ significantly between the groups. All of the procedures involving animals were conducted in accordance with the institutional animal welfare guidelines of the Institutional Animal Care and Use Committee (IACUC) of the Chung Shan Medical University.

### Tumor immunohistochemistry (IHC)

Paraffin embedded squamous cell carcinoma and paired non-cancerous tissue sections (4-μm) on poly-L-lysine-coated slides were deparaffinized in xylene and rehydrated in alcohol. Endogenous peroxidase activity was blocked with 3% H_2_O_2_. The antigen was retrieved by heating at 100°C for 20 min in 10 mM citrate buffer (pH 6.0). After antigen retrieval, slides were washed with PBS and incubated with anti-Ki67, anti-LC3, and anti-mouse or anti-rabbit immunoglobulin G (IgG) antibodies for 2 h at room temperature. After washing in PBS, slides were incubated with an horseradish peroxidase (HRP)/Fab polymer conjugate for another 30 min. The sites of peroxidase activity were visualized using 3,3′-diamino-benzidine tetrahydrochloride as a substrate. Gill Hematoxylin Solution II (MERCK, Darmstadt, Germany) was utilized as the counterstain. All specimens were deparaffinized and stained with hematoxylin and eosin (H&E) which was used as a light counterstain.

### Statistical analysis

Statistically significant differences were calculated using the Student's *t*-test (Sigma-Stat 2.0, Jandel Scientific, San Rafael, CA, USA). A *p* value < 0.05 was considered to be statistically significant. Values represent the means ± standard deviation and the experiments were repeated three times (*n* = 3).
