# Correction: Dehydroandrographolide, an iNOS inhibitor, extracted from Andrographis paniculata (Burm.f.) Nees, induces autophagy in human oral cancer cells

**DOI:** 10.18632/oncotarget.15541

**Published:** 2017-02-20

**Authors:** Ming-Ju Hsieh, Chiao-Wen Lin, Hui-Ling Chiou, Shun-Fa Yang, Mu-Kuan Chen

**Present**: Due to an error in proofreading, figure 8A is incorrect.

**Corrected**: The proper figure 8A is shown below. The authors sincerely apologize for this oversight.

**Figure 8:**
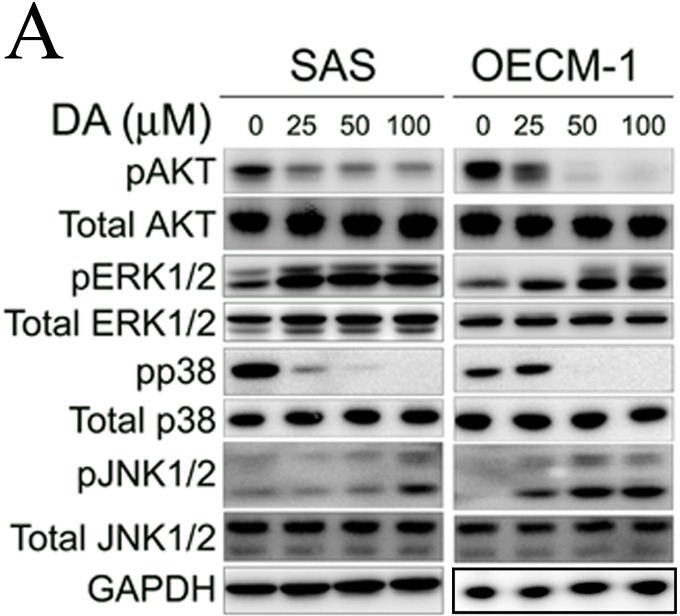
Effect of Akt and MAPK pathway on DA induces cell autophagy processes A. SAS and OECM-1 cells were treated with DA (0–100 μM) for 48 h and then subjected to western blotting for Akt, ERK1/2, p38 and JNK1/2 with GAPDH acting as an internal control.

Original article: Oncotarget. 2015; 6(31):30831-30849. doi: 10.18632/oncotarget.5036.

